# Perioperative Management of Interscalene Block in Patients with Lung Disease

**DOI:** 10.1155/2013/986386

**Published:** 2013-11-28

**Authors:** Eric S. Schwenk, Kishor Gandhi, Eugene R. Viscusi

**Affiliations:** Department of Anesthesiology, Jefferson Medical College, Suite 8490, 111 South 11th Street, Philadelphia, PA 19107, USA

## Abstract

Interscalene nerve block impairs ipsilateral lung function and is relatively contraindicated for patients with lung impairment. We present a case of an 89-year-old female smoker with prior left lung lower lobectomy and mild to moderate lung disease who presented for right shoulder arthroplasty and insisted on regional anesthesia. The patient received a multimodal perioperative regimen that consisted of a continuous interscalene block, acetaminophen, ketorolac, and opioids. Surgery proceeded uneventfully and postoperative analgesia was excellent. Pulmonary physiology and management of these patients will be discussed. A risk/benefit discussion should occur with patients having impaired lung function before performance of interscalene blocks. In this particular patient with mild to moderate disease, analgesia was well managed through a multimodal approach including a continuous interscalene block, and close monitoring of respiratory status took place throughout the perioperative period, leading to a successful outcome.

## 1. Introduction

Impaired lung function has traditionally been considered a relative contraindication to interscalene plexus block (ISB). ISB has been shown to cause ipsilateral hemidiaphragmatic paresis virtually 100% of the time [1, 2] with significant decreases in several pulmonary measurements [1]. Knowledge of the potential complications is critical, even if they occur rarely. At the same time, opioids impair respiratory function and should be minimized if lung function is tenuous [3]. The elderly in particular are sensitive to the depressant effects of anesthetics and medications that cause muscle weakness [4], especially opioids. Excellent postoperative analgesia, therefore, is a key component in the prevention of postoperative pulmonary complications in this population.

## 2. Case Description

The patient was an 89-year-old woman, American Society of Anesthesiologists Physical Status 3, with hypertension, hypothyroidism, and a 58-pack-year history of smoking who five years prior had undergone a left lung lower lobectomy for cancer. She was scheduled to undergo a right total shoulder replacement for worsening degenerative disease and pain. Pulmonary function testing performed 17 months prior to surgery revealed a FEV_1_/FVC ratio of 0.68, indicating mild obstructive disease, and a diffusion capacity (DLCO) of 9.5 mL/mm Hg/min, indicating a moderate gas transfer defect. Physical examination revealed clear lung fields bilaterally and a short hyomental distance on airway exam. Preoperative pulse oximetry on room air revealed an oxygen saturation of 100%. The patient and her family wished to proceed with surgery only under regional anesthesia after consulting with her primary care physician. After discussion of the risks and benefits of regional anesthesia, including the possibility of impaired lung function, pneumothorax on the operative side postoperatively, and mechanical ventilation postoperatively, the patient agreed to perform surgery under a continuous interscalene nerve block (CISB) with light sedation.

**Figure 1:**
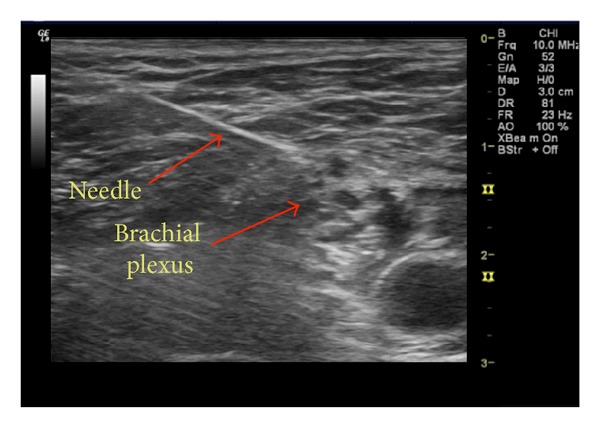
Ultrasound image of low interscalene block.

The block was performed using continuous ultrasound guidance (GE Logic E, Wauwatosa, WI) and nerve stimulation (B. Braun, Bethlehem, PA). An in-plane, posterior approach technique was utilized for needle insertion and visualization based on the preference of the anesthesiologist performing the procedure (Figure 1). A total of 30 mL of ropivacaine 0.5% was injected incrementally after negative aspiration to the area adjacent to the C5 and C6 nerve roots and a 20 g multiorifice peripheral nerve catheter (B. Braun Medical Inc., Bethlehem, PA) was inserted 5 cm beyond the needle tip. Sensory block was confirmed with decreased pinprick sensation in the C5 and C6 dermatomes. The patient was given a propofol infusion of 15–25 mcg/kg/min for sedation with oxygen via nasal cannula and surgery proceeded uneventfully.

In the postoperative anesthesia care unit (PACU) the patient had excellent analgesia. Intravenous fentanyl and morphine were available but she requested no rescue opioids or other medications. Close postoperative monitoring was continued on a surgical ward with frequent pulse oximetry measurements. Analgesia consisted of a multimodal regimen including CISB with ropivacaine 0.2% running at 8 mL/h without a demand function and ketorolac, aspirin, and pregabalin, with a morphine PCA for breakthrough pain. She consumed the equivalent of morphine 17.5 mg IV for breakthrough pain during the first 24 h postoperatively. She reported no side effects and denied significant dyspnea while the catheter was in place, receiving no more than 2 L/min of oxygen via nasal cannula as a precautionary measure during her admission. She used her incentive spirometer multiple times per day. She was discharged home on postoperative day no. 2 after the continuous interscalene catheter had been removed.

## 3. Discussion

### 3.1. Pulmonary Function Changes after ISB

Although ISB has traditionally been relatively contraindicated in those with decreased pulmonary function, we presented a case of an elderly woman with prior partial lung resection who experienced a successful outcome through minimizing opioids and close postoperative monitoring. Urmey and McDonald [1] demonstrated that multiple indices of lung function, including forced vital capacity (FVC), forced expiratory volume in one second (FEV_1_), and midexpiratory flow rate, are depressed when an ISB is performed. These findings have been subsequently confirmed by others [2, 5, 6]. Such changes are mostly due to the ipsilateral hemidiaphragmatic paresis that occurs with ISB, which likely persists for greater than four hours [2] and in one study extended to beyond eight hours after block [5]. The affected hemidiaphragm, in fact, will move in a paradoxical (i.e., cephalad) fashion after ISB in many patients [2]. There is some evidence that partial compensation by the contralateral hemidiaphragm may occur [6], however, which may explain why some patients with mild respiratory impairment can tolerate ISB without difficulty.

Phrenic nerve function is affected by the presence of a CISB, even after the primary block has been resolved. Pere and colleagues [5] demonstrated that some patients will have persistent impairment of diaphragm function for the duration of the continuous infusion. The implication is that sending an ambulatory patient with compromised respiratory function home with a continuous catheter could create a dangerous situation and should probably be avoided. The catheter for the patient described here was removed before discharge.

Pneumothorax following ISB is another consideration that should be discussed. Although many believe pneumothorax is less likely to follow ISB than supraclavicular block, and a recent prospective registry of more than 1,100 brachial plexus blocks (ISB and supraclavicular blocks) reported no pneumothoraces [7], caution must still be exercised. Several recent case reports document the occurrence of pneumothorax after ISB [8, 9]. Such a complication may go unnoticed in a healthy patient but could have serious consequences in someone with underlying lung disease or prior lung surgery. A pneumothorax could have been devastating for this patient, as it would have further decreased the lung area for gas exchange, potentially to a critical level, given her preexisting moderate gas exchange defect. Extra caution and discussion of this specific risk of ISB should precede its performance in a patient with prior lung resection or compromise for any other reason.

Finally, the long-term consequences of interscalene blocks are rarely discussed, but a recent case series [10] describing 14 patients who experienced long-term phrenic nerve paresis after ISB must be taken into context and factored into each case. These patients required surgical intervention to restore respiratory function. Unlike the patient in this case, all 14 patients were overweight or obese males.

### 3.2. Effects of Posterior versus Anterolateral Approach for the Interscalene Block

The anesthesiologist performing the block used an in-plane, posterior approach in which the entire needle is visualized, theoretically providing increased safety, more precise needle positioning, and avoidance of the surgical field [11]. This was chosen based on the preference of the anesthesiologist. However, the only prospective trial comparing the two approaches concluded that the anterolateral (out-of-plane) approach provided more pain-free time in the recovery room and easier catheter placement [12]. The issue remains unresolved, but, as it applies to this patient, evidence is lacking to support either technique reducing pulmonary complications.

### 3.3. Effects of Digital Pressure during Interscalene Block on Pulmonary Function

Digital pressure above the level of the ISB has been studied as a technique used to decrease the spread of local anesthetic to the phrenic nerve. Despite initial enthusiasm, this has been shown repeatedly to be ineffective [13–15].

### 3.4. Effects of Reducing Local Anesthetic Volume or Concentration on Pulmonary Function

Several investigators have studied the effects of decreasing the local anesthetic volume on hemidiaphragmatic paresis and other respiratory parameters. The results have been inconclusive, with some reporting an improvement in pulmonary function [16–18] and others finding that the diaphragm remains impaired [2, 6].

Studies examining the effects of using dilute local anesthetic solutions suggest that doing so may decrease some of the unwanted respiratory side effects [19, 20]. However, duration of analgesia would likely be shorter and potentially require the addition of a continuous catheter to provide adequate analgesia.

### 3.5. Comparison of Risks and Benefits

It has been shown that FVC, FEV_1_, and total lung capacity are reduced after lung lobectomy [21, 22], which for this patient resulted in mild obstructive disease and moderate gas exchange defect. Sengul et al. [23] found that pulmonary compensation after lower lobectomy in particular is achieved by expansion of the contralateral lung. A pneumothorax on the right (surgical) side for this patient, therefore, could have been especially deleterious. However, these concerns must be weighed against the pain and its detrimental effects on recovery. The benefits of adequate analgesia extend past the immediate postoperative period, as poorly controlled perioperative pain can lead to delayed hospital discharge and chronic pain syndromes [24].

Opioids remain an option but their unwanted side effects, in particular respiratory depression, limit their effectiveness in patients with compromised lung function. Specifically, opioids impair the diaphragm and thoracic muscles, decreasing functional residual capacity and leading to atelectasis [3]. A multimodal analgesic approach that includes regional analgesic techniques, nonsteroidal anti-inflammatory drugs (NSAIDs), acetaminophen, and opioids as rescue agents is ideal. The risks of ISB must be weighed against the potential respiratory effects of larger doses of opioids. For the intraoperative management, the decrease in functional residual capacity [25] and the atelectasis [26] that often occur under general anesthesia must be considered. Finally, some data have shown that regional anesthesia may reduce the risk of postoperative cognitive dysfunction in the elderly when compared to general anesthesia [27].

In summary, this 89-year-old woman was a motivated patient who understood the risks involved. Despite having mild to moderate lung disease, this patient was fairly well compensated and symptom-free on the day of surgery. Although this was reassuring, the potential for respiratory complications was nevertheless present. We believed that the benefits of regional anesthesia outweighed those of general anesthesia, taking the physiologic changes, patient preferences, and our own preferences into account. Through close observation in the PACU and continuing on the surgical ward, her respiratory status was maintained and clinically significant dyspnea and hypoxia were avoided. We believe this was a result of maximizing nonopioid agents and minimizing the consumption of opioids, encouraging incentive spirometer use, and close monitoring for any change in respiratory status.
